# Predicting Successful Aging in a Population-Based Sample of Georgia Centenarians

**DOI:** 10.1155/2010/989315

**Published:** 2010-09-14

**Authors:** Jonathan Arnold, Jianliang Dai, Lusine Nahapetyan, Ankit Arte, Mary Ann Johnson, Dorothy Hausman, Willard L. Rodgers, Robert Hensley, Peter Martin, Maurice MacDonald, Adam Davey, Ilene C. Siegler, S. Michal Jazwinski, Leonard W. Poon

**Affiliations:** ^1^Genetics Department, University of Georgia, Athens, GA 30602, USA; ^2^Tulane Center for Aging and Department of Medicine, Tulane University Health Sciences Center, New Orleans, LA 70112, USA; ^3^Institute of Gerontology, University of Georgia, Athens, GA 30602, USA; ^4^Department of Food and Nutrition, College of Family and Consumer Sciences, University of Georgia, Athens, GA 30602, USA; ^5^Survey Research Center, Institute for Social Research, University of Michigan, Ann Arbor, MI 48106, USA; ^6^The College of Saint Scholastica, Duluth, MN 55811, USA; ^7^Gerontology Program, Iowa State University, Ames, IA 50011, USA; ^8^School of Family Studies and Human Services, Kansas State University, Manhattan, KS 66506, USA; ^9^College of Health Professions, Temple University, Philadelphia, PA 19122, USA; ^10^Department of Psychiatry and Behavioral Sciences, Duke University, Durham, NC 27710, USA

## Abstract

Used a population-based sample (Georgia Centenarian Study, GCS), to determine proportions of centenarians reaching 100 years as (1) survivors (43%) of chronic diseases first experienced between 0–80 years of age, (2) delayers (36%) with chronic diseases first experienced between 80–98 years of age, or (3) escapers (17%) with chronic diseases only at 98 years of age or older. Diseases fall into two morbidity profiles of 11 chronic diseases; one including cardiovascular disease, cancer, anemia, and osteoporosis, and another including dementia. Centenarians at risk for cancer in their lifetime tended to be escapers (73%), while those at risk for cardiovascular disease tended to be survivors (24%), delayers (39%), or escapers (32%). Approximately half (43%) of the centenarians did not experience dementia. Psychiatric disorders were positively associated with dementia, but prevalence of depression, anxiety, and psychoses did not differ significantly between centenarians and an octogenarian control group. However, centenarians were higher on the Geriatric Depression Scale (GDS) than octogenarians. Consistent with our model of developmental adaptation in aging, distal life events contribute to predicting survivorship outcome in which health status as survivor, delayer, or escaper appears as adaptation variables late in life.

## 1. Introduction

**Figure 1:**
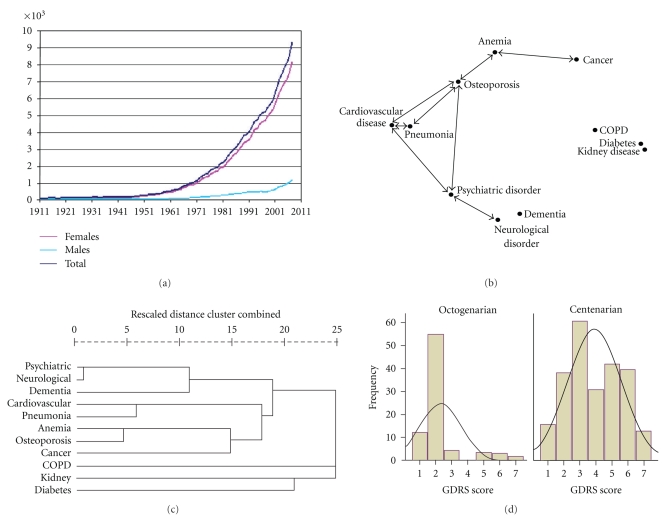
(a) Exponential growth of Centenarians in England over the last century [2]. (b) Network of morbidity in centenarians in the GCS [3] using lifetime prevalence of 11 most common chronic diseases in the GCS. All pairwise associations that were not significant by an exact test were set to zero, and the remaining significant (with *α* = 0.05) pairwise correlations (*r*_xy_) between chronic diseases *x* and *y* were all positive. These significant pairwise positive associations were graphically rendered using the distance 1-*r*_xy_ and multi-dimensional scaling [9] to compute coordinates for the chronic diseases. The coordinates were then graphed as a network [10]. COPD denotes chronic pulmonary obstruction disease. Chronic and acute pneumonia were not distinguished in the medical questionnaire. (c) Average linkage [11] was used to compute a dendrogram independently relating the 11 chronic diseases based on their pairwise correlations {*r*_xy_}. (d) The distribution of GDRS scores [12] in centenarians is compared with the distribution of GDRS scores in 80 octogenarians as a control group. A GDRS score of 4–7 is indicative of dementia, a score of 3, of mild cognitive impairment, and a score of 1-2, as unaffected.

With the expected lifespan of humans increasing at a rate of 2.5 years per decade [1], it will not be uncommon for individuals born in developed countries in this decade to live into the next century (Figure 1(a)) [2]. This observation poses a substantial challenge to health care and other entitlement systems because chronic diseases, which are the cause of 60 percent of deaths worldwide, often dictate the conditions of our later lives. Here we develop a means to predict the health-related survivorship outcomes of centenarians entering the next century.

The GCS obtained a population-based sample of 244 centenarians and near-centenarians (98 years old or older) and 80 octogenarians [3] through voter registration roles, and sampling from a complete list of nursing homes (NH) and personal care homes (PCH) in a 44-county area of northeast Georgia [3]. The GCS sample was drawn from counties in 4 strata, which were compared for sample characteristics with the 2000 US Census in Table 1. There is a reasonable agreement between the 2000 Census and the sample in the GCS with a slight under-representation of African-Americans. Sampling weights were developed to match sample characteristics with the 2000 Census profile (see Supplement). Statistical analyses below were performed with and without these weights with no change in conclusions. To address appropriately our own successful aging, we first need to answer the following questions: at 100 years of age, (i) will we be cognitively intact? (ii) what will be our emotional state? (iii) how likely is it that we will be coping with a chronic disease [4]? (iv) what chronic diseases will we need to cope with? (v) when will we experience a chronic disease?

## 2. Materials and Methods

The recruitment rate (i.e., the percentage of those participating out of those contacted) was 67.2% for centenarians and 46.0%, for octogenarians. Data on GCS participants were collected under Institutional Review Board approval and Human Subjects consent and are deposited in the Georgia Centenarian Database [5]. In contrast to Phase 1 and Phase 2 of the GCS, centenarians in Phase 3 here spanned a wide range of functional capacity from being bedbound in a nursing home to living independently in the community [3]. Demographic and medical histories of GCS participants were collected onto computer generated questionnaires and completed in 4, ~2 hour interviews with GCS participants. Sources for answers on the health questionnaire were participants, their legal proxy, care professionals in NHs or PCHs, and medical charts at NHs or PCHs. The health questionnaire included medical history, current problems (such as, bedbound status, assistive devices, and restricted activity days), medications/oxygen, physical examination (including vital signs, skin fold/Arm Circumference and hand grip, hearing, vision, fine motor testing, lower leg extension, foot sensory, weight/height, and shoulder flexion and vision test), EPESE and PPME tests of functional capacity (including standing balance, 8-foot walk, chair stands, step-up, and Bed Mobility), gross physical abnormalities, global assessment of physical health, and GDS [6]. International Disease Classification 9 (ICD-9) provided a framework for constructing the health questionnaire. More details on data collected have been previously given [3]. The instruments available at http://qa.genetics.uga.edu/ were then scanned into a Teleform database [5], checked, corrected, and verified and saved as individual pdf images for loading into the Georgia Centenarian Database [5]. A serious issue in centenarian studies is age validation [7]. The GCS employed internationally-established criteria in age verification [8]. The principal guideline is that chronological age must be validated by convergent multiple and creditable sources and public records, such as birth and marriage certificates of the individuals as well as their offspring and relatives to create a consistent chronology. Driver's licenses, Social Security documents, census records, as well as death records of offspring are used. 

 To validate medical histories on centenarians, nonfasting blood samples were drawn by a skilled phlebotomist as previously described [3]. From these blood samples, glycated hemoglobin (HbA1c) and hemoglobin levels were assessed by a clinical diagnostic laboratory (LabCorp, Inc., Burlington, NC) and used for diagnosis of diabetes and anemia, respectively. Dementia status was also cross-validated by a combined neuropathological and clinical consensus report on 66 centenarians, who consented to neuropsychology followup and brain donation postmortem [3]. Results are to be reported elsewhere.

A multinomial logistic response model was fitted to the study data [13] with SPSS Software at (http://www.spss.com/). The response was whether or not a centenarian is a survivor (S), delayer (D), escaper (E), or other (O) with probability *π*_*ij*_, where *i* indexes all levels of the independent variables, sex, race, institutional status, education, body mass index, use of tobacco (i.e., the *i*th subpopulation), and the 11 indicators of chronic diseases found in Figure 1(b) and *j* = S, D, E, or O. Using a “[“ for closed and “)” for open as a standard mathematical notation, a survivor of chronic diseases is a centenarian who first experiences chronic diseases in earlier years from [0,80). A delayer is a centenarian who only first experiences chronic diseases late in life from [80,98), and an escaper is a centenarian who only encounters chronic diseases at the very end of life at 98 or older. These definitions of survivor, delayer, and escaper differ slightly from those in [4] by using a cutoff of 98 (instead of 100) for the escaper category and by using a different list of chronic diseases in Figure 1(b). For the multinomial response model here, we use all 11 chronic diseases in Figure 1(b) to define survivor, delayer, or escaper. More restrictive definitions for particular disease classes are also examined in the Results and Discussion (see Table 3).

The log-likelihood is product-multinomial and proportional to

(1) $$l\left(B\right)=\overset{m}{\underset{i}{\sum }}\overset{J}{\underset{j}{\sum }}n_{ij}\ln\;\;\left(\pi_{ij}\right).$$

The cell probabilities *π*_*ij*_ are determined by the independent variables and given by

(2) $$\pi_{ij}=\frac{\exp\;\;\left(x_{i}^{\prime }\beta_{j}\right)}{1+\sum_{k=1}^{J-1}\exp\;\;\left(x_{i}^{\prime }\beta_{k}\right)},$$

where *x*_*i*_′ is the vector of observations on the *i*th subpopulation and *β*_*j*_ is the vector of regression coefficients for the *j*th response. The reference response is *J*. The Other (O) category arose when there were missing data, and the reference response was survivor (S). The model was fitted by the method of maximum likelihood [13].

## 3. Results and Discussion

*Will we be cognitively intact?* The prevalence of cognitively intact centenarians is still being debated. There is broad variation (27%–100%) in the literature [3] about the prevalence of dementia in centenarians partially due to differences in assessment methods and partially due to age, gender, race, educational attainment ratios, and sampling techniques (e.g., convenience samples versus population-based samples). Sampling methods can be vital to the results when one considers that the functional capacity of centenarians varies dramatically from one extreme of a Nobel Laureate [15], Dr. Rita Levi-Montalcini, who serves currently in the Italian Senate, to someone needing the support of a nursing home. A Global Deterioration Scale (GDRS) battery [12] was administered to GCS participants. Dementia is scored when an individual receives a score of 4–7 on the GDRS. As shown in Figure 1(d), approximately half of centenarians (57%) scored as having dementia, while the majority of octogenarians were cognitively intact. 

**Figure 2:**
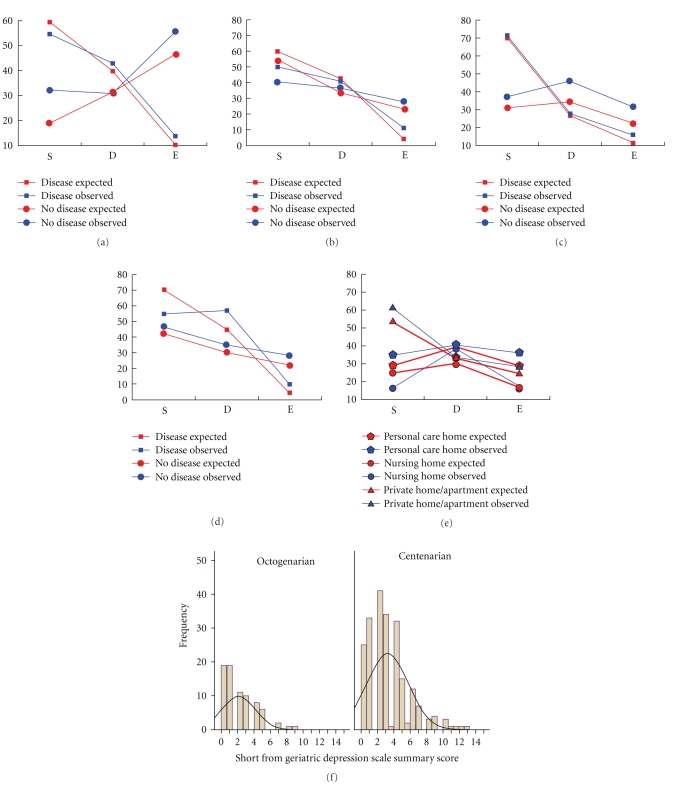
The factors of cardiovascular disease (a), cancer (b), pneumonia (c), psychiatric disorders (d), and living arrangement (e) determine the fraction of survivors, delayers, and escapers [4] among centenarians. The observed (in blue) and expected proportions (in red) track each other in that the logistic multinomial model well predicts the outcome of a being a survivor (S), delayer (D), or escaper (E) [4]. In panels (a)–(e), a square indicates the presence of a disease and a circle, the absence of a disease. (f). Centenarians were higher (with mean of 3.21 and standard deviation of 2.56) on the Geriatric Depression Scale (GDS) [6] than the control group of octogenarians (with mean of 2.13 and standard deviation of 2.62). The proportion (15.5%) of centenarians with GDS from 6–15 is significantly different from that proportion (4.6%) in octogenarians (*Z* = 3.13, *P* < .001).

*What will be our emotional state once we reach 100?* Based on a health history questionnaire, centenarians do not appear to experience more depression, anxiety, or psychoses relative to octogenarian controls (Table 2). This is surprising because the prevalence of dementia, which is associated with psychiatric and neurological diseases (Figure 1(b)), is significantly higher in centenarians versus octogenarians (Figure 1(d)). To validate concurrently these findings, centenarians and the control group were compared on the Geriatric Depression Scale (GDS) [6] short form (Figure 2(f)). The short form GDS has a range from 1 to 15, with 6–10 suggestive of depression and 11–15 almost always indicative of depression. In Figure 2(f), there is a significant difference by a *t*-test (*t* = 3.68, df = 296, *P* < .001) in the mean GDS between centenarians and octogenarians. What this means is that while there is no evidence for a difference in lifetime prevalences of psychiatric disorders (other than dementia) in Table 2, there is a subclinical difference in level of depression.

*How likely is it that we will be coping with a chronic disease?* There are three mutually exclusive avenues to 100 in this study [4]. We can be survivors of chronic diseases in earlier years from 0–80. Alternatively, we can be delayers and only first experience chronic disease late in life from 80–98, or we can be escapers and only encounter chronic disease at the very end of life at 98 or older. The avenue by which we achieve 100 years of age depends on the chronic disease encountered (Table 3c). There are significantly different outcomes with respect to cardiovascular disease and cancer when it comes to how we reach 100. For example, cardiovascular disease (i.e., congestive heart failure, myocardial infarction, high blood pressure, peripheral vascular disease, stroke, transient ischemic attack (TIA), or any other heart problems) has a more even distribution across the three avenues, while centenarians with cancer are mostly escapers (73%). Both categories, cardiovascular disease and cancer, aggregate across 8 or more distinct forms of cardiovascular disease or cancer.

We compared the avenues to 100 in the New England Centenarian Study (NECS) and GCS. These two studies differ in population, sampling methods, and chronic disease categories. Despite these differences, the NECS [4] percentage of survivors, delayers, and escapers is 38, 42, and 19%, respectively in Table 3a, which is similar (not significantly different by an exact test [14] in Table 3a) to the corresponding percentages in the GCS of 43, 36, and 17%, respectively. Matching to the NECS chronic disease, selection did not change this outcome (Table 3b). When all chronic diseases in Figure 1(b) are considered, the fraction of escapers is relatively small among centenarians (17–24% in Table 3). This percentage is also very similar to a Danish nearly complete longitudinal, 1905 birth cohort study of successful aging in 40,000 Danes with 19% escapers [16]. 

Based on these survivorship outcomes and their apparent stability across three studies, we can make predictions about the cohort beginning in the year 2060. To do so we add one more category, the attritor, who never survives to be a centenarian. For the original cohort yielding the GCS centenarians, only about 0.0002 became centenarians now, and 0.9998 are attritors. For the cohort originating in 2060 [1], we would expect that ~0.5 will live to be centenarians, that is will not be attritors. Our prediction on the challenge to entitlement systems in the next century is that the percentages of escapers, delayers, survivors, and attritors will be 8.5%, 18%, 21.5%, and 50%, where for example 8.5% = 100 × (0.5) × (0.17). Other factors affecting this prediction are now discussed.

Both genetic and environmental factors influence successful aging [18–21], particularly the successful outcome of no chronic diseases until very late in life. Other factors that might influence the three avenues to 100 include a participant's sex, race, institutional status (i.e., living in the community, PCH, or NH), education, chronic diseases experienced, body mass index, and smoking. A multinomial response model was fitted [13] stepwise to data on GCS centenarians in which the response was a centenarian classified as survivor, delayer, escaper, or other (missing data for 8 centenarians), and the independent variables were sex, race, institutional status, education, tobacco use, body mass index, and 11 chronic diseases in Figure 1(b) including dementia. The results in Table 4 and Figures 2(a)–2(e) display the significant factors (by likelihood ratio test with significance level of 0.05) to be lifetime prevalence of psychiatric disorder(s), cardiovascular disease, cancer and past prevalence of pneumonia as well as institutional status (i.e., living in the community, PCH, or NH). Goodness of fit for this multinomial response model is adequate (Pearson *χ* ^2^ = 59.5 with df = 78). The Cox and Snell pseudo-*R* ^2^ was computed [13] as ~0.39. The influence of these five factors on the probability of being a survivor, delayer, or escaper is shown in Figures 2(a)–2(e) as well as the graphical fit of the model to the data. Being at risk for cardiovascular disease, cancer, and psychiatric disorders decreases the chance of becoming a delayer or escaper (Figures 2(a), 2(b), and 2(d)). Institutional status drops out as significant if the response categories of survivor and other are collapsed together.

Distal variables, such as level of education, mother's education, childhood health, and number of major life events (marriage, divorce, loss of spouse, etc.) for each centenarian can have an impact on current health [22]. Only for a subset of GCS participants (97) were all of these distal variables available. The number of major life events was significant (*P* < .014) in a stepwise fitting procedure to predict a centenarian's outcome as survivor, delayer, or escaper (see Materials and Methods). Goodness of fit remained adequate with the addition of this distal variable (Pearson *χ* ^2^ = 75.9 with df = 90). The Cox and Snell pseudo-*R* ^2^ increased to 0.51 with this one additional independent variable, indicating distal variables do help to predict survival outcome.

*What chronic diseases will we need to cope with?* Individuals in the GCS were characterized by their history of chronic diseases based on medical histories and an extensive battery of psychosocial tests. The positive associations (edges) between the 11 most frequent chronic diseases (nodes) of centenarians are graphically rendered by multidimensional scaling and network software [9, 10] (Figure 1(b)). There appear to be two clusters of chronic diseases: one resembles a multicausal cluster having such common causes/determinants as smoking, imbalanced nutrition, sedentary lifestyle, and includes cardiovascular disease, pneumonia, osteoporosis, anemia, and cancer; and a second cluster including dementia, psychiatric disorders, and neurological disorders. Those diseases that are connected are significantly associated by an exact test [14] (*α* = 0.05) of diseases X and Y, in which centenarians are classified by disease status for diseases X and Y in a 2 × 2 table. The two clusters were independently generated by clustering using average linkage [11] (Figure 1(c)).

**Figure 3:**
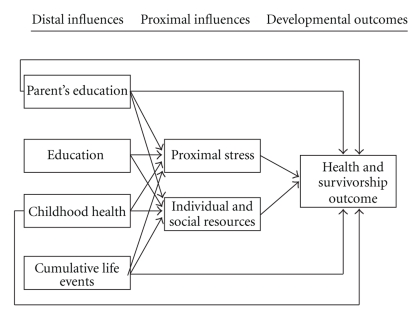
Developmental Adaptation: The influence of distal variables (e.g., cumulative life events, parents' education, education, and childhood health) on adaptational outcomes in very late life.

The fact that dementia does not correlate with cardiovascular disease may at first sight seem surprising. This is, however, not the first time this observation has been made. In the Nun study [23], dementia did not necessarily correlate with neuropathology. There are in fact at least two forms of dementia, Alzheimer's disease and vascular dementia [24] with the latter being much less prevalent and correlated with vascular disease. In centenarians, only 12% of the cases of dementia were reported as vascular dementia [25]. The low prevalence of vascular dementia could be one explanation for the lack of correlation with dementia as scored by the GDRS in Figure 1(d), which does not distinguish the two forms of dementia. As discussed in the next section, cancer and cardiovascular disease have an earlier presentation than dementia. It is possible that other factors, such as coping mechanisms with stress and lifestyle (Figure 3), can also intervene to affect dementia status at 80 years of age and beyond and weaken the association further. 

In some cases, the chronic condition reported in medical histories could be independently validated. For example, diabetes can be independently validated by glycated Hemoglobin (HbA1C) levels (a cutoff >7% is indicative of diabetes), and a declaration of diabetes by HbA1c levels is highly associated with reports from medical histories by Fisher's Exact test in both centenarians and octogenarians (*P* < .005). As a second example hemoglobin (Hb) levels (in grams/deciliter or g/dL) were determined on centenarians and octogenarians. When GCS participants are classified as anemic using a cutoff of 12 g/dL in females and 13 g/dL in males, an exact test of association between these two classifications of GCS participants for anemia is *P* = .05. There is underreporting of anemia from medical histories relative to anemia defined from participant Hb levels. 

A summary of the prevalent chronic diseases among centenarians is summarized in Table 5. Cardiovascular disease and dementia are the most prevalent chronic diseases among centenarians. For some conditions, such as osteoporosis, dementia, and psychiatric disorders, the prevalence differs significantly between the sexes.

*When will we experience a chronic disease?* For centenarians, the development of chronic diseases varies by type. It is clear from Figure 1(d) that the prevalence of dementia rises sharply between the ninth and eleventh decade of life. A similar question is addressed about age of onset for cancers and cardiovascular disease among centenarians in Table 4b. Cancers (with the exception of skin cancers) tend to have early onset in the eighth decade (seventies) of life, and as a consequence we saw no difference in their frequency between octogenarians and centenarians (Table 4b). In contrast, cardiovascular disease has a later onset in the ninth decade of life in GCS (Table 4a). This presents a puzzle for why there is no difference in proportions between centenarians and octogenarians in cardiovascular disease (Table 4b). To correct for right censoring and to address this puzzle, we performed a separate Cox-regression analysis [17] on age at onset for cancer and cardiovascular disease as a function of the covariates used in the multinomial response modeling with race being a significant factor in a stepwise Cox-regression analysis of cancer age at onset. The effect of the Cox regression was to shift the estimated means of cancer and cardiovascular disease earlier to 60 and 77 years of age, respectively. The earlier mean onset corrected for right censoring then explains why in Table 4b there is no difference in lifetime prevalence of cardiovascular disease between octogenarians and centenarians. In that GCS is a cross-sectional study, there is a need to validate this temporal pattern of barriers to successful aging being cancer and then cardiovascular disease in cohort studies [16, 18].

In summary, our data show that there are 11 chronic diseases that centenarians are likely to experience late in life. These diseases fall into two morbidity clusters, one involving such diseases as cardiovascular disease, cancer, anemia, and osteoporosis, and another cluster associated with dementia. These chronic diseases pose three major barriers to successful aging. In their sixties centenarians are at risk for cancer. In their seventies they are at risk for cardiovascular disease. In their eighties and beyond they are at risk for dementia. Approximately half (43%) of the centenarians did not experience dementia. Approximately 17% of the GCS centenarians escaped chronic disease till near the end of their life, while 36% delayed the onset into the 80's and 90's and 43% survived chronic diseases acquired earlier in life (<80 years of age). These proportions of escapers, delayers, and survivors serve as predictions for the 2060 cohort with median life expectancy of 100 in this population. The caveats on such a prediction include no cohort effects (i.e., war, disease, major health advance, or changes in the predictors in Figure 2) beyond those leading to the increase in life expectancy of 2.5 years per decade. With the exception of dementia, mental health status did not differ between centenarians and octogenarians, although levels of depressive symptomatology appeared to be higher on the GDS scale in centenarians than octogenarians. Consistent with our model of developmental adaptation [22] (Figure 3), distal life events contribute to predicting survivorship outcome. The morbidity classification put forth by the NECS [4] and current health status are critical adaptation variables in very late life.

## Supplementary Material

The supplement gives a detailed description of the dual sampling frame of the study using voter registration rolls and a list of nursing homes and personal care homes. Weights are developed for octogenarians, near-centenarians, and centenarians to bring the sample into alignment with the target population. The weight assigned to each member of a sample can be thought of as the estimated number of individuals in the target population that are represented by that individual. The weights developed depend on post-stratification. They are designed to bring the sample into agreement with the target population on the basis of county of residence, age, gender, race, and type of residence. The iterative procedure for determining these weights is described in the supplement. The use of weights is evaluated relative to an unweighted procedure using mean square error.

## Figures and Tables

**Table:** (a) Age Distribution, 2000 Census versus GCS Participants

	Participants		2000 Census	
Age	Number	Percent	Number	Percent
98	61	25%	362	30%
99	48	20	275	23
100–104	126	52	526	42
105+	9	4	81	6
TOTAL	244	100%	1244	100%

**Table:** (b) Gender Distribution, 2000 Census versus Participants

	Participants		2000 Census	
Gender	Number	Percent	Number	Percent
Male	37	15%	237	19%
Female	207	85	1007	81
TOTAL	244	100%	1244	100%

**Table:** (c) Race Distribution, 2000 Census versus Participants

	Participants		2000 Census	
Race	Number	Percent	Number	Percent
Black	52	21%	397	32%
Non-Black	192	79	847	68
TOTAL	244	100%	1244	100%

**Table 2:** The lifetime prevalences of psychiatric disorders other than dementia in centenarians versus octogenarian control group in GCS [3] do not differ.

Disease*	Octogenarians (%)	Centenarians (%)	Total #
Dementia	13 (14%)*	136 (57%)*	321
Depression	14 (17.5%)	36 (14.8%)	324
Anxiety	5 (6.3%)	17 (7.0%)	324
Psychosis	1 (1.3%)	6 (2.5%)	324
Total	33	185	324

*The row categories are not mutually exclusive, but dementia tends to be positively associated with psychiatric disorders (Figure 1(b)). The association of Dementia with age (Control versus Centenarian) is significant with *P* < .00001 by Fisher's Exact test [14] for a 2 × 2 table, but the associations of Depression, Anxiety, and Psychosis individually with age (Control versus Centenarian) are not significant with *P* > .05. If we combine mental health across Depression, Anxiety, and Psychosis and test for association with age (Control versus Centenarian) by Fisher's Exact test [14] for a 2 × 2 table, the association of the aggregate variable indicating Depression, Anxiety, or Psychosis with age is not significant. The percents reported in this table are among 80 octogenarians or 244 centenarians.

**Table:** (a) Avenue to 100 in GCS (*N* = 244) and NECS studies (*N* = 424)

Avenue	Survivor (%)	Delayer (%)	Escaper (%)
GCS	43	36	17
NECS	38	42	19

Outcomes (columns) are not significantly different by exact test (*P* = .27) [14].

**Table:** (b) Avenue to 100 in GCS and NECS studies when matched on chronic diseases

Avenue	Survivor (%)	Delayer (%)	Escaper (%)
GCS	35	35	24
NECS	38	42	19

Outcomes (columns) are not significantly different by exact test (*P* = .14) [14].

**Table:** (c) Avenue to 100 in GCS dependent on disease category

Avenue	Survivor (%)	Delayer (%)	Escaper (%)
Cardiovascular disease	24	39	32
Cancer	11	14	73

Outcomes (columns) are significantly different (*P* < .0001) between disease categories by an exact test [14].

**Table:** (a)

Disease	Average Age at Onset	*n*	Average Age at Onset from Cox regression	*n*
Cancer*	78 ± 3	22	60 ± 0.5	238
Cardiovascular	83 ± 1	181	77 ± 0.3	226

*Many cancers are absent, and skin cancers were excluded because of their later onset. The *n* above differs from the totals in **(b)** due to missing data.

**Table:** (b)

Disease	No cancer	Cancer	Total
Octogenarians	58	22 (28%)	80
Centenarians	171	73 (30%)	244
Total	229	95 (29%)	324

A Fisher's exact test [14] is not significant at *α* = 0.05, nor is a *z*-test, significant, with *z* = 0.90 on the proportions, 0.28 and 0.30.

**Table:** (c)

Disease	No Cardiovascular	Cardiovascular	Total
Octogenarians	19	61 (76%)	80
Centenarians	48	196 (80%)	244
Total	67	257 (79%)	324

A Fisher's exact test [14] is not significant at *α* = 0.05, nor is a *z*-test, significant, with *z* = 1.80 on the proportions, 0.76 and 0.80.

**Table 5:** Lifetime Prevalence of chronic diseases among centenarians in the GCS.

	Prevalence		Prevalence	
Chronic Disease	Male (%)	Female (%)	Pooled over sexes (%)	Number of centenarians
cardiovascular	27 (73)	169 (82)	196 (80)	244
dementia	15 (41)	121 (60)*	136 (57)	240^$^
pneumonia	15 (41)	90 (43)	105 (43)	244
cancer	14 (38)	59 (29)	73 (30)	244
osteoporosis	1 (3)	58 (28)***	59 (24)	244
psychiatric	2 (5)	45 (22)*	47 (19)	244
anemia	5 (14)	37 (18)	42 (17)	244
diabetes	3 (8)	18 (9)	21 (9)	244
kidney	7 (19)	11 (5)**	18 (7)	244
neurological	4 (11)	11 (5)	15 (6)	244
COPD	2 (5)	2 (1)	4 (2)	244

*P < .05 by Fisher's Exact Test of disease associated with sex [19]; **P < .01 by Fisher's Exact Test of disease associated with sex [19]; ***P < .001 by Fisher's Exact test of disease associated with sex [19].

^$^Four centenarians had missing data.
