# Supplementary material for: Predicting Successful Aging in a Population-Based Sample of Georgia Centenarians
Source: Curr Gerontol Geriatr Res. 2010 Sep 14;2010:989315. doi: 10.1155/2010/989315 (PMC2946582; doi:10.1155/2010/989315)
Supplement: Supplementary file 1 — The supplement gives a detailed description of the dual sampling frame of the study using voter registration rolls and a list of nursing homes and personal care homes. Weights are developed for octogenarians, near-centenarians, and centenarians to bring the sample into alignment with the target population. The weight assigned to each member of a sample can be thought of as the estimated number of individuals in the target population that are represented by that individual. The weights developed depend on post-stratification. They are designed to bring the sample into agreement with the target population on the basis of county of residence, age, gender, race, and type of residence. The iterative procedure for determining these weights is described in the supplement. The use of weights is evaluated relative to an unweighted procedure using mean square error. [file 989315.f1.pdf]

Sample Design for Georgia Centenarian Study, and Creation of Weights

Willard L. Rodgers[1]

April 16, 2009

[1] Survey Research Center, Institute for Social Research, University of Michigan, Ann Arbor, MI 48106

Abstract

The third phase of the Georgia Centenarian Study is a multidisciplinary, population-based study of centenarians and near-centenarians living in the northern part of Georgia. It was designed to identify, locate, and gather information from all centenarians in the target population. For this purpose, a dual frame was implemented. To identify individuals in skilled nursing facilities and personal care homes, a list of all such places in the designated counties of Georgia was assembled and a subset of those institutions was asked to provide a list of their residents aged 98 and older. To identify those living in the community, the Voter Registration List maintained by the Georgia Secretary of State was scanned to find all individuals with addresses in the target area who were age 98 and older at the beginning of the data collection period. Difficulties in getting the cooperation of the staffs of facilities, and in obtaining current telephone numbers for those on the Voter Registration List, as well as the substantial proportion of those who were identified who were unable or declined to participate, resulted in a participation rate estimated to be about 20 percent, but the target sample size was met, with 244 centenarians and near-centenarians. In addition, a sample of 80 octogenarians was obtained primarily from the Voter Registration List, and samples of 100 each in their third, fourth, fifth, and sixth decades were obtained through a random digit dialing procedure. Weights have been developed to provide improved correspondence between the samples of centenarians and octogenarians and the target populations.

Introduction

Centenarians are one of the fastest growing age groups in the U.S. and world populations, their number in the U.S. is estimated to have increased by 51 per cent over the decade between 1990 and 2000 and is projected to increase even faster in the current decade (Kestenbaum and Ferguson, 2005). They nevertheless remain a relatively rare population, constituting on the order of 1 out of every 10,000 Americans.[2] This means that the usual methods for identifying a random sample from the general population, such as area probability sampling and random digit dialing techniques, are not applicable to the population of centenarians; the costs involved in making thousands of calls, whether by telephone or by knocking on doors, to find one household in which a centenarian resides, would far exceed the costs of data collection once an eligible person had been located. In addition, more than a third of centenarians do not live in households, but instead in nursing homes or assisted living facilities that are typically not included in the sampling frames for surveys of the general population.

Despite (indeed, because of) their rarity, centenarians are of considerable scientific interest. Studies of centenarians have revealed a wide range of risk factors related to the probability of survival to extreme old age, including demographic, psychological, socio-economic, genetic, and nutritional habits and characteristics. To permit generalization beyond the specific group of centenarians who are included in a research project, it is important to be able to link the sample to a larger population, and ideally this requires that every member of the target population have a known, non-zero probability of being included in the sample.

A variety of methods have been used to identify and recruit centenarians for studies. Several studies conducted in Europe and Asia have taken advantage of registration systems that are maintained for administrative or other purposes. The Okinawa Centenarian Study has drawn its sample using family and

---

[2] According to the 2000 US Census, there were 50,354 individuals aged 100 or more in a total population of 281 million, or about 1 centenarian per 5578; see http://factfinder.census.gov/servlet/DTTable?_bm=y&-geo_id=01000US&-ds_name=DEC_2000_SF1_U&-mt_name=DEC_2000_SF1_U_PCT012 However, Kestenbaum and Ferguson (2005) used Medicare records cross-checked against Social Security applications and early census records to obtain an estimate of just 32,920 centenarians on Jan. 1, 2000, or 1 centenarian per 8535 in the total population as enumerated in the 2000 Census.

resident registration systems that were instituted in Japan as early as 1872 (Willcox et al., 2008). The Swedish Centenarian Study (Samuelsson et al., 1997) identified and tried to recruit all new centenarians (specifically, those who were within six months after their 100th birthdays, over a five year period) from a registry of residents in the southern part of the country. The Longitudinal Study of Danish Centenarians (Andersen-Ranberg, Vasegaard, and Jeune, 2001) identified all centenarians living in Denmark who reached their 100th birthdays during a 14 month period, using the Civil Registration System. The Italian Multicentric Study on Centenarians (Capurso et al., 1997) selected a random sample of centenarians identified in a census that covered 29 percent of the population of Italy. The Korean Centenarian Study (Choi et al., 2003) identified centenarians living in the Seoul metropolitan area and in three provinces, using a national registry of centenarians maintained by the Ministry of Health and Welfare.

Even where registration systems exist, however, they are not always available for research purposes, or are not convenient to use. For example, a study in Japan recruited its centenarian sample from volunteers (Chan, Suzuki, and Yamamoto, 1997). In the U.S., registration systems do exist that cover a large proportion of the elderly population: Social Security and Medicare. In the past, the Medicare Enrollment Database has been used to draw samples of elderly populations, but the availability of that system has been greatly limited in recent years. For this reason, population-based studies of centenarians in the U.S. are rare. For example, the New England Centenarian Study generated its sample of centenarians in part from local censuses conducted in eight towns in the suburban Boston area (Perls et al., 1999), in part from mass mailings, and in part from responses to reports of the study in the media (Terry, Sebastiani, Andersen, and Perls, 2008).

The first phase of the Georgia Centenarian Study (GCS) collected data from 137 centenarians who were identified through referrals from a wide range of local and stage agencies, churches, and mass media; all were living in the community, including old age homes and life care communities as well as in private households, and all were capable of completing in-home interviews. The second phase of the GCS reinterviewed survivors from the first phase the study. Given the procedure by which the

participants were identified, the target population was limited to community residents in reasonably good physical and cognitive health, and it was not possible to assign probabilities of selection to members of that population.

The third phase of the Georgia Centenarian Study was a multi-disciplinary, population based study of centenarians and near-centenarians residing in the northern part of Georgia. Its goals are described more fully at the study website (http://www.geron.uga.edu/research/centenarianstudy.php) and in other publications (Poon et al., 2007; Martin et al., 2006; Johnson et al., 2006), but in brief they were to provide psychological, physiological, genetic, and nutritional information from and about people aged 98 and older. In addition, comparative data were obtained from and about samples of people residing in the same geographic area in their third, fourth, fifth, sixth, and ninth decades of life.

The purposes of this paper are twofold: First, to describe the target populations for the third phase of the GCS and procedures used to select sample of those populations; and second, to describe the development of weights that allow analysts to obtain unbiased (or at least less biased) estimates of population parameters from the sample data.

Target populations

The primary target population for the third phase of the GCS consisted of individuals who were 98 years of age or older at the beginning of one of a series of field periods. The population included those living in nursing homes and assisted living facilities as well as those in private households. The geographic coverage consisted of 44 counties in Georgia, with a total population of 5,003,311, including 1,244 age-eligible residents, according to the 2000 Census.[3] Seventy percent (871/1244) of the centenarians and near-centenarians resided in the Atlanta metropolitan area; this is a lower proportion than the overall population, of whom 79 percent (3,945,450) lived in the Atlanta metropolitan area.

[3] If Kestenbaum and Ferguson's (2005) estimate of the U.S. population of centenarians in 2000 is correct, the Census overcounted centenarians by over 50 percent (50354/32920 = 1.53). If the same degree of overestimation held in northern Georgia, the size of the target population was approximately 800.

For comparative purposes, samples were also drawn from five younger target populations. These populations were defined by residence in the same geographic area (the 44 counties of northern Georgia), and by age at the time of the relevant data collections. The age ranges for these populations, and their size as of the 2000 Census, were as follows:

20-29 years: 776,304

30-39 years: 885,971

40-49 years: 774,282

50-59 years: 533,425

80-89 years: 83,208

Sample design for centenarians and near-centenarians

As noted in the introduction, designs that have been developed for selecting samples of the general population, including area probability samples and random digit dialing techniques, are not appropriate for rare subpopulations such as centenarians. Other studies of centenarians have made use of census and administrative files that are compiled for other purposes. Such files, if they are sufficiently comprehensive of the target population, allow random samples to be drawn and contacted in straightforward and relatively inexpensive manners. Such a list is not currently available for research purposes in the United States. The decennial censuses would be a possibility (though because of high mortality rates, decreasingly efficient as time since the last census increases) were it not for strict rules that do not permit individually-identified information to be released until seventy years after each census. The Social Security system is another possibility, especially with the implementation of new rules requiring children to be registered for their parents to be able to claim them as dependents on their income tax returns, but as with census data, regulations prevent the use of the Social Security frame for research purposes. A third registration system in the U. S. is the Medicare Enrollment Database maintained by the Centers for Medicare and Medicaid Services (CMS), a file that is estimated to include about 97% of the U.S. population aged 65 and older. This has been used in numerous research projects to draw samples of

elderly populations, and in fact the originally proposed design for identifying centenarians in the third phase of the GCS was based on use of this source. This design had to be abandoned, however, when there was a change in policy of CMS with respect to the provision of names of Medicare enrollees for research purposes just at the time that we were ready to begin the process of data collection. When it became clear that this change would be permanent and that this made it impractical to implement the proposed sample design, we developed a revised sample design.

The revised sampling plan had two components. The first started with a census of all skilled nursing facilities (SNFs) and personal care homes (PCHs) located in the 44-county area and continued with the identification of all residents of a sample of those facilities who were age 98 and older. The second component relied on the Voter Registration List maintained by the state of Georgia, again across the entire 44-county area, and used the date-of-birth information contained on that list to identify individuals who were recorded as being age 98 and older.

Each of these components has limitations. By starting with a list of facilities rather than of individual centenarians, we inserted another set of gatekeepers – the staffs of these facilities whom we asked to provide names of all centenarians and near-centenarians in residence – who may because of privacy concerns or simple lack of time be reluctant to comply. Moreover, the Voter Registration List does not include all members of the target population, though it does include a much higher proportion of those living in private households than of those in facilities who are better covered by the first frame.

Implementation of the sampling procedures

To obtain the power needed to achieve the objectives of the GCS, the primary goal of the sampling and data collection procedures was to complete a series of interviews with 240 centenarians. The total number of centenarians in the 44 counties was estimated to be approximately 600, based on the 2000 Census; or about 400 if the count for centenarians in those counties is inflated to the same degree as Kestenbaum and Ferguson (2005) estimate for the U.S. as a whole. That is, to reach the needed sample size would require finding and gaining the cooperation of at least 40 per cent, and perhaps as high as 60

percent, of all eligible individuals. Given the limitations of the sampling frames, the need to obtain the cooperation of the staff in facilities and of concerned family members as well as the centenarians themselves, and the substantial proportion of centenarians who have physical health or cognitive limitations that would decrease their likelihood of participating, obtaining data from even 40 percent of all eligible individuals seemed optimistic. One option that was considered was increasing the geographic area, but even if the target population were to have been expanded to the entire state of Georgia the expected count would only have increased by about 50 percent (from 607 to 906, based on the 2000 Census), and at the same time would have resulted in a large increase in field costs to pay for travel time and expenses of the data collection staff, and would have required establishing additional facilities where blood samples could be processed within a short enough time after being gathered.

For this reason, the decision was made to expand the age range of the target population to include “near centenarians” – those age 98 and 99 – as well as those aged 100 and older. Based on Census counts, this was expected to double the size of the target population.

In order to achieve control over the number of centenarian and near-centenarian participants and maximize the proportion of those respondents who were over age 100, the 44 counties were divided into four strata, defined so as to be mostly contiguous and with approximately the same population of centenarians as estimated from the 2000 Census. The field periods for these strata were scheduled to run successively, with each lasting approximately six months.

The target population for each of the four strata was defined as persons residing within the geographic boundaries of the stratum who were age 98 or older by the beginning of the field period for that stratum. To implement the sample design, lists were obtained of all SNFs and PCHs in each of the 44 counties, and the number of beds in each of those facilities. In this way, a total of 161 SNFs with 19,592 beds and 649 PCHs with 13,820 beds were identified. A random half of the 109 PCHs with 7 – 25 beds, and a random quarter of the 362 PCHs with 6 or fewer beds, were selected. The names were requested of all centenarians in all SNFs and in all large PCHs (defined as those with more than 25 beds), and in the

selected smaller PCHs. In addition, in the first two strata, the names of all near-centenarians were requested in a random quarter of the selected SNFs and PCHs. However, based on the experience in identifying and recruiting centenarians in these facilities in the first two strata, the decision was made to request the names of the near-centenarians (as well as the centenarians) in all of the selected SNFs and PCHs in the remaining two strata to allow the desired number of participants to be obtained.[4]

Distribution of participating centenarians and near-centenarians on demographic variables

The distributions of the 244 centenarians and near-centenarians who fully participated in the series of data collections on four demographic variables are shown in the left hand pair of columns of Table 1 (parts of which are reproduced, as Tables 1a – 1c, in the body of the paper). The next pair of columns shows the distribution of the target population on these variables as estimated from the 2000 Census. The final column provides a rough estimate of the percentage of eligible individuals in the target population who participated in the study.

Over half (126) of the participants were in the age range of 100 to 104, [5] a considerably higher proportion than the population in the same geographic area at the time of the 2000 Census, when only 42 percent (526) were in that age range. There were correspondingly fewer participants age 98 (25 percent) or 99 (20 percent) than observed in the population (29 and 22 percent, respectively). The final column shows the implied participation rates for those in each age group, with a considerably higher rate for those age 100-104 than for those of other ages. This at least in part is due to the fact that initially we selected 98 and 99 year olds from only a fraction of the SNFs and PCHs from which we selected centenarians. The second panel in Table 1 indicates that a slightly smaller proportion of the participants were male (15 percent) than was reported in the 2000 population (19 percent). A much more sizeable discrepancy is observed with respect to race: only a fifth (21 percent) of the participants was identified as African

[4] Details about the data collection procedures are provided in McCarthy, Grier, Johnson, Poon, and Reynolds, in press).
[5] The Census Bureau does not publish tables that distinguish single years of age for individuals over age 99, and this rule also applied to the special tabulations that the Bureau produced at our request.

American, as compared to a third (32 percent) of the 2000 population.[6] The implication is that the participation rate was almost twice as high for non-Black as for Black centenarians, as shown in the final column. The fourth panel shows the distributions across four geographic strata; the estimated response rates are lower in strata 3 and 4, which correspond fairly closely to the Atlanta metropolitan statistical area (MSA), than in the two other strata.

Large differences are also observed in the final panel of Table 1, which shows the distribution on type of residence, based on special tabulations of the 2000 Census that we requested from the U.S. Census Bureau.[7] Whereas 62 percent of the population lived in households in 2000, only 38 percent of the participants did so. Correspondingly, the participation rate is estimated to be less than half for those living in households as it is for those in facilities. The underrepresentation of the household population, relative to those in nursing homes and group quarters, likely is the result primarily of two factors. First of these factors is that the voting rolls do not include all individuals, and perhaps this is especially true of very old individuals, since the voter roll is supposed to be purged after several elections have passed with no voting activity. The second factor is difficulty in contacting many of the eligible individuals on the Voter Registration List. That list includes name, address, and date of birth, but no telephone number. Interviewers attempted to obtain a telephone number for each eligible voter, first by using a reverse directory available via the internet, then by calling telephone company information services, but often without success, perhaps because the voter had moved, or perhaps because the telephone number was

---

[6] The race of the participants was self-reported. In the 2000 Census, household informants were asked to designate the race of each member, and were allowed to choose multiple categories for each person. For the special tabulations requested from the Census Bureau, we asked that everyone for whom Black (more specifically, the category labeled "Black, African Am., or Negro") was mentioned, either alone or in combination with other racial categories, be distinguished from all others. (We also requested that a distinction be made in the non-Black category between those who mentioned White and all others, but since less than 2 percent of the population of the 44 counties aged 98 and older were in that category, we have not retained that distinction in these tables. All of the participants in our study identified themselves as either Black or White.)

[7] Information about type of residence of centenarians is not included in publications based on the 2000 Census. Because this was expected to be a critical variable for evaluating the sample and for the development of weights, we requested a set of special tabulations be generated, in which type of residence was tabulated against age, gender, and race. Because of rounding requirements for special tabulations, the counts in these tables do not exactly match published tables. All counts in the special tabulations were rounded to the nearest 10, in accordance with rules approved by the Disclosure Review Board: <http://www.census.gov/population/www/cen2000/sptabs/disclosure.html>. This has only minor consequences. For example, the total count of individuals in the 44 counties, age 98 and older, is 1240 according to the special tabulations, compared to a total of 1244 in published tables. Where direct comparisons could be made (on gender and age distributions), the differences were no more than one percentage point.

listed in the name of another household member. If no telephone number could be found, no further attempt was made to locate the voter.

Table 1: Distribution of Centenarians on Demographic Characteristics, Participants vs. 2000 Census

	Participants		2000 Census		Participation Rate
	Number	Percent	Number	Percent	
Age					
98	61	25%	362	30%	17%
99	48	20	275	23	13%
100-104	126	52	526	42	24%
105+	9	4	81	6	11%
Gender					
Male	37	15%	237	19%	16%
Female	207	85	1007	81	21%
Race					
Black	52	21%	397	32%	13%
Non-Black	192	79	847	68	23%
Geographic stratum					
1	80	33%	313	25%	26%
2	30	12	133	11	23%
3	79	32	491	39	16%
4	55	23	307	25	18%
TOTAL	244	100%	1244	100%	20%
Type of residence					
Household	93	38%	770	62%	12%
Institution	103	42	310	25	33%
Group Quarters	48	20	160	13	30%
TOTAL	244	100%	1240	100%	20%

About two thirds of the participants who were not household residents were in nursing homes, and the remainder were in Personal Care Homes; this distribution matches that observed in the 2000 Census, when two thirds of the non-household residents (25 percent of all age 98 and older) were classified as institutionalized and the remainder as living in non-institutional group quarters.

Reasons to use weights

The comparisons of the sample distributions with the 2000 Census distributions on the demographic variables, as shown in Table 1, indicate that some parts of the target population are much better represented in the sample than are others. These discrepancies can be expected to introduce biases

into estimates of population estimates that are derived from the sample data, not only for these demographic variables but for any characteristics that are related to them – in other words, for almost any variable of interest.

To reduce such biases, it is common practice to use weights in the analysis of sample data: weights that adjust the sample to bring it into alignment with the target populations at least with respect to characteristics for which the population distribution can be estimated from an independent source. For this reason, we have developed a weight variable that we recommend be applied in analyses, in particular for the purpose of estimating univariate parameters such as population totals, means, and proportions. Before we describe the specific procedure we used to develop and evaluate that weight, we provide a brief overview of the rationale that underlies the use of sample weights.

The weight assigned to each member of a sample can be thought of as the estimated number of individuals in the target population that are represented by that individual. In general, scientific studies collect data from samples that are a small fraction of the target population – for example, if every 100th name was selected from a list of the entire target population, each sample member can be thought of as representing 100 members of the population, and a weight of 100 could be assigned to the data collected from each individual to estimate population totals.

Often the sample design is more complex than the one just described, where each member of the population had an equal probability of selection. For example, some segments of the population – say, those with a particular risk factor for a disease, or those in particular minority groups – may be sampled at a higher rate than the rest of the population. Such differences in sampling probability would be taken into account by assigning weights that are, again, the inverse of each individuals sampling probability; those in subgroups selected with higher probability would have smaller weights than those chosen with lower probability..

In addition to differences in sampling probability, there may be differences in response rates for different segments of the population, or differences in success in contacting different types of people.

These differences, as well, are often taken into account through weights, under the assumption that those sample members in a subgroup who fail to participate in the study are more like the members of their subgroup who did participate than they are like other members of the population. For example, if males selected for a study have a response rate of 50% whereas females have a response rate of 75%, the males would be assigned a weight factor of 2, whereas females would be assigned a weight factor of 1.33, under the assumption that non-responding males are more like responding males than they are like responding females; and similarly for the non-responding females. If there were also differences in sampling probability, the weights developed to compensate for those differences would be multiplied by the weight factor to compensate for non-response.

Finally, weights may be adjusted to bring the weighted distribution of the sample into line with known population distributions; this is known as post-stratification. In general, this would be the final step in the development of sample weights, after differences in sampling probability and differences in contact rates and response rates have been taken into account.

Development of weights for centenarians

The procedure used to develop weights for the centenarians differ in some respects from the general procedure for defining sample weights. First it is important to note that, unlike the typical sample survey, the goal of this study was, with some minor exceptions, to identify all centenarians in a specific geographic area; to contact each of those identified; and to recruit as many of them as possible as participants in the data collection activities. The minor exceptions were, first, that random samples were taken from small PCHs to reduce the large upfront costs of contacting and securing the cooperation of many of these small facilities, most often only to learn that they had no resident centenarians; and second, initially residents age 98 and 99 were identified in only a random fourth of the SNFs and PCHs in which those age 100 and older were identified – but this differentiation between near-centenarians and actual centenarians was later abandoned. The initial step in the process of developing the weights, as described below, is intended to take account of the sampling of small PCHs, and therefore of their residents.

Second, we made no attempt to take account specifically of differences in the response rate for different parts of the population, because of practical considerations. Obtaining the cooperation of a sample member depended on a long and often complex series of steps, especially for those in nursing homes and personal care facilities. An appropriate administrator had to be identified in each facility, and then the study had to be explained to that person. A request was then made of that administrator for a list of all residents age 98 and older (or, in some of the initial cases, those age 100 and older). Often this request was not met directly; instead, the administrator sometimes agreed to contact a relevant family member of each such resident and to ask them to contact study staff if they were willing for them to approach the resident, and only then could we identify those residents, try to contact them, and ask them if they would participate.

In the case of household residents, it was often found that the information on voting rolls was incorrect or out of date, and thus many of the individuals on the voter rolls who were designated as eligible (age 98 or older) were never contacted. Many of these were likely ineligible because they had died, or had moved out of the designated geographic area, but it was impossible for us to estimate what proportion of the selected cases were actually eligible and therefore we could not estimate the participation rate.

The creation of weights, therefore, depended primarily on post-stratification. That is, the steps of the procedure described below is designed to bring the weighted sample distribution into close agreement with the target population with respect to the five characteristics (county of residence, age, gender, race, and type of residence) that we could obtain from the 2000 Census. Partly because we did not have a five-way cross-tabulation of the Census data, but primarily because the number of cells in such a detailed cross-tabulation would have been very large and the number of empty or sparse cells almost equally large – and therefore the weights would have been highly variable – we instead achieved this approximation through an iterative (or raking) process. That is, we adjusted first on one of the five characteristics

(substratum), then readjusted on cross-tabulation of successive pairs of the remaining four characteristics; and we repeated these steps until we achieved a stable set of weights from one iteration to the next.

The steps that we followed in creating weights for the sample are described in detail in a working paper (Rodgers, 2008, downloadable from the GCS website[8]). Here we give an overview of the procedure.

We began by creating a weight that took account of the rate at which smaller personal care homes were sampled: all participants were assigned an initial weight of 1, except those in PCHs with 25 or fewer beds. Those in PCHs with 7-25 beds were assigned a weight of 2, to account for the fact that only half of such facilities were asked to provide the names of any centenarian or near-centenarian residents. (Those in PCHs with fewer than 7 beds would have been assigned a weight of 4, but as it happens, there were no such participants.)

We then made successive adjustments to the initial weight to adjust the weighted sample size to the 2000 Census counts on each of five variables: geographic substratum (sets of counties of residence), gender, age, race, and type of residence. Since we could not do so simultaneously for all five of these variables, we instead did so for one or two variables at a time, and repeated this cycle of adjustments until the weights assigned to each participant stabilized across cycles. At the first of these steps, the participants were classified into the 4 strata that were used to sequence the data collection efforts, and then further classified into a total of 11 substrata, each consisting of one or more of the 44 counties. The weights of the participants living in each of these substrata were multiplied by a constant such that the sum of the weights for participants in each substrata was equal to the total number of centenarians (and near centenarians) in those same counties as enumerated in the 2000 Census. Then, at the next step the participants were classified into six cells defined by the cross tabulation of their gender and their type of residence, and the weights of the participants in each of these six cells were multiplied by a constant such that the sum of the revised weights was equal to the Census count in that cell. The third step adjusted the

[8] http://www.geron.uga.edu/research/centenarianstudy.php

weights of participants within each of eight cells defined by the cross-classification of age category and type of residence, and the last step in each cycle adjusted the weights for cases in cells defined by the cross-classification of their race and type of residence. Then the whole series of adjustments was repeated several times until the weights for participants changed only minimally from one cycle to the next.

The set of weights generated by the procedure just described provides the best possible correspondence between the distribution of the participants and the distribution of the centenarians enumerated in the 2000 Census on the five demographic characteristics. We will refer to this as Weight A (W_A). In addition we created three variations on this basic weight, and in the next section of the paper we will compare these weights and decide which one has the most desirable properties. The reason to consider these alternative weights is that not only do we want to use weights that minimize biases in sample estimates of population parameters, we also want to use weights that minimize the sampling variability of the sample estimates. The more variability there is in a set of weights across a sample, the more sampling variability there is in sample estimates.[9] The second set of weights, call these W_B, that we will consider is derived from the first simply by trimming the largest and smallest weights – specifically, weights were trimmed at the 5th and 95th percentiles (that is, the smallest 5 percent of the weights were all given the value of the 5th percentile, and the largest 5 percent were all given the value of the 95th percentile). The third set of weights, call these W_C, truncated the initial weights more drastically by trimming them at the 10th and 90th percentiles. The fourth and final set of weights, call these W_D, started with W_B and then put these weights through the iterative (raking) procedure used to generate the original (W_A) weights. Finally, to make the four sets of weights directly comparable each set of weights was normalized by multiplying each respondent's weight by a constant such that the sum of the set of weights (across all respondents) was equal to the estimated population size (i.e., 1244).

---

[9] For example, if we were to select two samples of the same size from the same population, one in which every member of the population has the same probability of selection, the other in which half of the population – say males – are selected with probability 1 in 100,000, the other half – females – are selected with probability 1 in 1,000, the estimates of population parameters such as means and proportions obtained from the second of these samples would have much more sampling variability than those obtained from the first sample.

Evaluation of the weights for centenarians

The distributions of the three different weights described above (W_A, W_B, W_C, and W_D) are compared in Table 2. By construction, the sum of each weight across all participants is set equal to the 2000 Census count for the population in the 44 counties aged 98 and older, which was 1244; and so all three weights have the same mean (5.098 = 1244/244). As intended, the truncated weights (W_B and especially W_C) have considerably less variance than do the untruncated weights (W_A); and the range of weights is cut almost in half by the truncation at the 5th and 95th percentiles (W_B), and by almost two thirds by the truncation at the 10th and 90th percentiles (W_C). The final set of weights, W_D, in which the truncated weights, W_B, were readjusted to improve the alignment of the weighted data with the demographic variables, seems to a large extent to have recreated the greater variance and greater range of the original weights (W_D vs. W_A).

Table 2: Comparison of Alternative Weights for Centenarians

Weight	Average	S.D.	Min.	Max.
W_A	5.098	5.065	0.308	30.947
W_B	5.098	4.166	0.662	16.136
W_C	5.098	3.557	0.947	11.798
W_D	5.098	4.959	0.520	30.097

The penalty exacted by the need to use weights can be expressed as the Weight Effect, or WEFF, which is defined as the square of the ratio of the standard error of the weighted mean to the unweighted standard error. This was calculated for each of the four weights for each of 67 variables; a summary is provided in Table 3. The average WEFF is larger than 2 for both W_A and W_D, and somewhat smaller for the truncated weights: at 1.67 for W_B and 1.45 for W_C. Another interpretation of the WEFF is that it is a measure of the reduction in the effective sample size because of the need to use weights to get unbiased estimates of population parameters (in this case, of population means). A WEFF of 2 indicates that the effective sample size for the estimation of the statistic is just half of the nominal sample size. Across the 67 variables examined, the use of W_A reduces the effective sample size by a factor of 1/2.229, or .449 – the average effective sample size, then, is 244/2.229 = 109.5. Use of W_B reduces the effective sample size

to 244/1.671 = 146.1; use of W_C reduces the effective sample size to 168.7; and use of W_D reduces it to 115.2.

Table 3: Average Weight Effect for Centenarians

	WEFF		
	**Average**	**Minimum**	**Maximum**
W_A	**2.229**	**0.681**	**8.278**
W_B	**1.671**	**0.624**	**3.696**
W_C	**1.446**	**0.623**	**2.358**
W_D	**2.118**	**0.656**	**7.473**

We are confronted with a set of alternative weights, and now we needed a basis for deciding which to select. In making that choice, we considered two factors: on the one hand, we wanted to minimize the biases in our sample-based estimates of population parameters; and on the other hand, we wanted to minimize the sampling errors in those estimates. With respect to the first criterion (the minimization of biases), we generally lack a gold standard – we do not know the population value for most parameters of interest. In the absence of such a gold standard, we used estimates based on W_A as the one we expected to have the least bias since it is that weight that does reproduce most faithfully the population distributions on the five characteristics where those population distributions can be directly estimated from the 2000 Census.

With respect to the second criterion (the minimization of sampling errors), we used the standard errors for sample means as obtained from a software package (Stata) that takes proper account of the weights (using the "svy: mean" command)

We then calculated a statistic that combines these two criteria: the mean square error (MSE) in the estimate of means for a set of study variables; and compare this statistic across five estimates of these means: the unweighted mean, and the mean calculated using each of the four weights we developed (W_A, W_B, W_C, and W_D). The preferred estimate varies across the variables we have considered. For some variables, it appears that the best estimate is the unweighted mean, while for others the best estimate is one based on one or another of the weights. But these MSE values are themselves subject to sampling

variability, and in any case it would not be practical to use different weights as tailored to specific analyses; we want to select a single weight that appears optimal across a wide range of analyses. For this reason, we aggregated the MSEs across a whole set of variables, and selected the weight that we concluded provides the best overall estimates of population characteristics.

The mean square errors in the estimates of population means were calculated for a set of 67 demographic and substantive variables, using these four weights as well as the unweighted estimates. To illustrate the procedure, the unweighted average age of the participants was 100.58 years, but this is somewhat of an overestimate compared to the Census, and this is reflected in weighted averages that range from 100.31 (W_B) to 100.40 (W_C). A penalty is paid when using weights, however, in the form of greater sampling variability as reflected in the higher standard errors of the weighted averages compared to the weighted average. For age, the standard error of the unweighted average is 0.13 years; this increases to 0.20 when the average is estimated using W_A; falls to 0.17 when using W_B; and increases back to 0.20 when using W_D. The mean square error, which combines the estimated bias with the sampling variability, is greatest for the unweighted estimate (0.239); lowest for the estimate based on W_B (0.18); and intermediate for the estimates based on W_A and W_D (0.20). Relative to the MSE for the estimate based on W_A, the MSE for the unweighted mean is 20% higher while the MSE for the estimate based on W_B is about 9% lower.

Scanning through these statistics for the 67 test variables revealed considerable variation. Sometimes the unweighted mean has the smallest MSE (e.g., for the number of hand taps, where the bias of the unweighted mean is small and the sampling variability is considerably larger for the weighted than for the unweighted estimates). Variation is also seen across the four weights – sometimes the MSE is smallest for W_A, other times for W_B, W_C, or W_D.

To allow an overall summary to be made across variables with different units and with different variances, the ratio was calculated of the MSE for the estimate obtained using a particular weight, W_w, to the standard error of the estimate using the untruncated weights, W_A. The average values of these relative

MSEs across the 67 variables examined are shown in Table 4. This indicates that, on average, the MSE in the unweighted estimates is about 27 percent higher than the standard error of the estimate obtained using W_A. The range is considerable: at one extreme the MSE of the unweighted mean is only about two-thirds of the S.E. based on W_A, while at the other extreme the unweighted MSE is six times as large as the weighted S.E. The averages of the MSEs obtained using the truncated weights, W_B and W_C, are smaller than that of the MSEs based on the untruncated weights; the average ratios are 0.939 and 0.944, respectively; again the ratio varies across the 67 variables, but not nearly as much as the ratio for the unweighted estimates, especially for W_B. The MSEs for the estimates based on the re-adjusted truncated weights, W_D, generally lie between those based on W_A and those based on W_B or W_C. For individual variables, the estimates based on W_B have smaller MSEs than those based on W_A in 56 of the 67 variables (76 percent); and also are generally smaller than the MSEs for the unweighted means (for 51 of the 67 variables) and than the MSEs for the means based on W_D (for 53 of the 67 variables). Based on this analysis, it appears, first, that the estimates from the four weights are approximately the same and that there are not large differences in their sampling variability; second, that estimates obtained using any of these weights are clearly preferable to unweighted estimates; and third, that the mildly truncated set of weights, W_B, tends to be superior to any of the alternative weights, at least for estimating mean values.

Table 4: Average Relative MSE for Centenarians

	**$MSE_w/S.E._6$**		
	**Average**	**Minimum**	**Maximum**
Unweighted	**1.267**	**0.655**	**5.957**
W_6	**(1.000)**	**(1.000)**	**(1.000)**
W_7	**0.939**	**0.727**	**1.155**
W_8	**0.944**	**0.669**	**1.382**
W_9	**0.982**	**0.915**	**1.082**

Specification of sampling procedures for octogenarians

As with the sample of centenarians, we used a stratified sample design, with the same four sets of counties, and with data collection in each stratum taking place in the same period as for the centenarians. Due to the much larger number of 80-year-olds than of centenarians on the voter rolls and the fact that we

did not need as many recruits, we randomly selected a sample of octogenarians for recruitment. Given the small sample size (eighty total participants) and the relatively small proportion of octogenarians who reside outside of households (about 15%), we specified a design that stratified both on the geographic strata based on county of residence, and on type of residence (household vs. non-household). A target was set of 12 participants (15% of the total) residing in SNFs or PCHs, and the remaining 68 participants in households.

For the household sample, lists of octogenarians (that is, individuals who had passed their 80th but not their 90th birthdays at the start of the field period for a particular stratum) and resident in that stratum were drawn from the voter registration file. We started the first and second strata with lists of 34 such names, on the assumption that half of those selected would be alive, could be contacted by telephone using address and telephone information included on the voter registration file, and would agree to participate in the study. It soon became clear that the assumption about the response rate among octogenarians was considerably over-optimistic. Additional samples of 34 octogenarians in each of the first two strata were generated. For the third stratum a sample of 68 octogenarians was provided; and for the final stratum a sample of 112 octogenarians was provided.

For the non-household sample, in each of the four strata we selected a subset of SNFs that were cooperative with the recruiters' requests for the names of centenarians, and asked each such SNF for the name of a resident who was an octogenarian and whose last name came closest, alphabetically, to a pair of randomly generated letters. Our intention was to identify a sufficient number of octogenarians to provide three participants who resided in a small subset of the SNFs that were selected for the sample of centenarians.

These procedures yielded the required number of octogenarian participants: that is, a total of 80 participants, 12 of whom (15%) were in SNFs. One participant selected from the voter registration list turned out to reside in a PCH.

Distribution of participating octogenarians on demographic variables

The distributions of the 80 octogenarians who fully participated in the series of data collections on four demographic variables are shown in the first pair of column of Table 5. The second pair of columns shows the distribution of the target population on these variables, based on the special tabulations of the 2000 Census that we requested from the U.S. Census Bureau.

Table 5 shows the distributions on demographic variables. Over half (45) of the participants were in the age range of 80 to 84, but this is a considerably lower proportion than the population in the same geographic area at the time of the 2000 Census, when 64 percent were in that age range. The proportion of the participants who were male was about the same in the sample (34 percent) as in the 2000 population (32 percent). Unlike the case for centenarians, the proportion of octogenarian participants who identified themselves as Black (18 percent) was only slightly lower than the proportion in the 2000 Census (20 percent). Given the stratified design, it is not surprising there is only a small difference in the distribution on type of residence is observed: of the 80 octogenarian participants, 67 (84 percent) lived in households, compared to 86 percent in the 2000 Census.

Table 5: Distribution of Octogenarians on Demographic variables, 2000 Census vs. Participants

	Participants		2000 Census	
	Number	Percent	Number	Percent
Age				
80-84	45	56%	53,280	64%
85-89	35	44	30,010	36
Gender				
Male	27	34%	26,330	32%
Female	53	66	56,960	68
Race				
Black	14	18%	16,270	20%
Non-Black	66	83	67,020	80
Residence				
Household	67	84%	71,980	86%
Non-household	13	16	11,310	14
TOTAL	80	100%	83,290	100%

Development and evaluation of weights for octogenarians

As with the centenarians, we developed and evaluated several alternative weights to improve the correspondence between the octogenarian participants and the target population of all octogenarians living in the designated 44 counties in Georgia. We take the 2000 Census data for octogenarians living in the designated geographic area as a reasonable substitute for the target population as it was over the period of data collection. We used the same procedures for developing these weights as those described earlier for developing the weights for centenarians. A detailed comparison of these weights is provided elsewhere. Based on our analysis, it appears, first, that the estimates from the four weights are approximately the same and that there are not large differences in their sampling variability; second, that estimates obtained using any of these weights are clearly preferable to unweighted estimates; and third, that the truncated and readjusted set of weights, W_D, tends to be slightly superior to any of the alternative weights, at least for estimating mean values.

Data availability

The data from the third phase of the GCS are archived in an integrated database system (Dai et al., 2006). The weights described in this paper (excluding those specific to Project 4) will be included in that database.

Summary

The rarity of centenarians, their physical and cognitive limitations, and the diversity of their living arrangements all create difficulties for collecting data that can be generalized to a population of centenarians. In the third phase of the Georgia Centenarian Study, the intention was to collect data from a sample of centenarians that would allow generalizations to be made to all centenarians in 44 counties of northern Georgia. A dual sampling frame was adopted; one designed to identify centenarians living in nursing homes and assisted living facilities, the other primarily to identify centenarians living in the community. To implement the first of these frames, a list was compiled of all skilled nursing facilities and personal care homes in the designated geographic area, and staff in those facilities were asked to

provide a list of all their residents who were age 98 and older. For the second frame, the Voter Registration list was used to identify individuals age 98 and older.

Problems encountered in implementing both of these frames sharply limited the proportion of centenarians who could be approached to seek their participation in the data collection activities. It was often difficult to make contact with appropriate staff persons at facilities, and when someone was found, they were often reluctant to release names of centenarians directly to the research staff, instead perhaps offering to contact next of kin to invite them to contact the research staff if they were willing to allow the resident centenarian to participate. With respect to the Voter Registration list, the primary problem was that the list did not contain a telephone number, so research staff had to try to find such a number from telephone directories, and for a substantial proportion of the registrants who were age eligible according to the list, no number was ever found and no further effort could be expended to try to contact those people. Moreover, among those for whom telephone numbers were found, substantial numbers turned out to be deceased, or to be age ineligible, having birth dates different from what was recorded on the voter list. These difficulties encountered in identifying centenarians, compounded with the more usual issue of some of those who were identified not being able or willing to participate in the extensive data collection activities, resulted in a participation rate that is estimated to be approximately 20 percent.

To compensate, at least in part, for imbalances that may exist between the 244 centenarians and near-centenarians who participated in the study and the target population estimated to be approximately 1244 in number, a weight variable was created that took into account five demographic characteristics of the sample. These characteristics – age, gender, race, type of residence, and county of residence – are also known for centenarians and near-centenarians who were enumerated in the 2000 Census, which we have taken as approximately equivalent to the target population of that age group at the time of the data collection. Four versions of the weight variable were created and compared to one another as well as to the unweighted analysis, seeking an optimal balance between minimizing the bias in sample means and proportions and the sampling variability of those sample estimates. This evaluation indicated that each of

the four weight variables provided better estimates than did unweighted analyses, and allowed us to recommend one of those four weights for use in analyses designed to estimate population parameters.

In addition to the sample of centenarians, data were also collected from control samples of octogenarians and of those age 20 – 59 living in the same region of Georgia. The sample of octogenarians was selected primarily by use of the Voter Registration List, and secondarily from lists of residents of skilled nursing facilities. Again, a weight variable was generated to improve the correspondence between the sample of octogenarians and the target sample as represented by all octogenarians enumerated for the region in the 2000 Census. The samples of younger age groups were identified through random digit dialing, and the objective of obtaining data from 100 participants in each decade of age from 20 – 59 was achieved.

We conclude with a cautionary note. In the GCS, as in any survey in which a substantial proportion of the sampled cases either cannot be contacted or are unwilling or unable to participate once contacted, there should be concern about how well the participants represent the target population. In most studies, very little is known about the non-participants, so an implicit if not explicit assumption is made that participation vs. non-participation is independent of study variables (for example, see Dillman et al., 2002). This assumption (that the data are missing completely at random, or MCAR) is often weakened to the assumption that participation vs. non-participation is independent of study variables within categories of the population (that the data are missing at random, or MAR). This is the assumption that underlies the argument for the use of weights such as those described in this paper: the population is categorized by demographic or other variables that are known from a census or other external source, and the participants are assigned weights to bring their weighted numbers into conformity with the target distributions. However, it is very possible that the assumption of randomness in participation, even in its weaker MAR form, is misleading in many studies, including the GCS. Non-participation may well be related to important study variables such as the physical health of centenarians and their cognitive abilities, even within categories defined by age, gender, race, county of residence, and type of living

arrangements. This possibility is more potent because of the fact that the participation rate is on the order of 20 to 30 percent, since the magnitude of a non-response bias on the estimation of population mean on a particular variable can be conceptualized as the product of the discrepancy in the average value between participants and non-participants times the non-participation rate.

References

Andersen-Ranberg, K., Vasegaard, L., and Jeune, B. 2001. “Dementia is not inevitable: A population based study of Danish centenarians.” **Journal of Gerontology: Psychological Sciences**, **56B**:P152-P159.

Capurso, A., Resta, F., D’Amelio, A., et al. 1997. “Epidemiological and socioeconomic aspects of Italian centenarians.” **Archives of Gerontology and Geriatrics**, **25**:149-157.

Chan, Y.-C., Suzuki, M., and Yamamoto, S. 1997. “National status of centenarians assessed by activity and anthropometric, hematological and biochemical characteristics.” **Journal of Nutritional Sciences and Vitaminology, 43**: 73-81.

Choi, Y.-H., Kim, J.-H., Kim, D.K., Kim, J.-W., Kim, D.-K., Lee, M.S., Kim, C.H., and Park, S.C. 2003. “Distributions of ACE and APOE polymorphisms and their relations with dementia status in Korean centenarians.” **Journal of Gerontology: Medical Sciences**, **58**:227-231.

Dai, J., Davey, A., Siegler, I.C., Arnold, J., and Poon, L.W. 2006. “GCSDB: an integrated database system for the Georgia Centenarian Study.” **Bioinformation**, **1**:214-219.

Dillman, D.A., Eltinge, J.L., Groves, R.M., and Little, R.J.A. 2002. “Survey nonresponse in design, data collection, and analysis.” Pp. 3-26 in Groves, R.M., Dillman, D.A., Eltinge, J.L., and Little, R.J.A.: **Survey Nonresponse**. New York, John Wiley & Sons.

Johnson, M.A., Davey, A., Hausman, D.B., Park, S., and Poon, L.W. 2006. “Dietary differences between centenarians residing in communities and in skilled nursing facilities: the Georgia Centenarian Study. ” **Age**, **28**:333-341.

Kestenbaum, B.M. and Ferguson, B.R. 2001. “Number of centenarians in the United States Jan. 1, 1990, Jan. 1, 2000, and Jan. 1, 2010, based on improved Medicare data.” In: **Living to 100 and Beyond** Monograph. The Society of Actuaries, Schaumburg, Illinois, U.S.A.

Martin, P., Rosa, G.d., Siegler, I.C., Davey, A., MacDonald, M., and Poon, L.W. 2006. “Personality and longevity: findings from the Georgia Centenarian Study.” **Age**, **28**:343-352.

McCarthy, E.K., Grier, K., Johnson, M.A. , Poon, L.W., and Reynolds, S. 200x. Recruiting the Oldest-Old for Gerontology Research: Lessons from the Georgia Centenarian Study.

Perls, T.T., Bochen, K., Freeman, M., Alpert, L., Silver, M.H. 1999. "Validity of reported age and centenarian prevalence in New England." **Age and Ageing**, **28**: 193-197.

Poon, L.W., Jazwinski, S.M., Green, R.C., et al. 2007. "Methodological concerns and pitfalls in studying centenarians: lessons learned from the Georgia Centenarian Studies." In Poon, L.W. and Perls, T.T. (Eds), **Annual Review of Geriatrics and Gerontology**, **27**: 231-264.

Samuelsson, S.-M., Alfredson, B.B., Hagberg B., Samuelsson, G., Nordbeck, B., Brun, A., Gustafson, L., and Risberg, J. 1997. "The Swedish Centenarian Study: A multidisciplinary study of five consecutive cohorts at the age of 100." **International Journal of Aging & Human Development, 45**:223-253.

Terry, D.F., Sebastiani, P., Andersen, S.L., and Perls, T.T. 2008. "Disentangling the roles of disability and morbidity in survival to exceptional old age." **Arch. Internal Med., 168**(3): 277-283.

Willcox, D.C., Willcox, B.J., He, Q., Wang, N., and Suzuki, M. 2008. "They really are that old: A validation study of centenarian prevalence in Okinawa." **Journal of Gerontology: Biological Sciences, 63**(4): 338-349.
